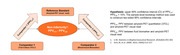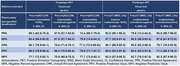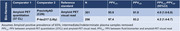# Evaluating the interchangeability of blood‐based biomarkers and amyloid‐PET for identifying patients with Alzheimer’s pathology

**DOI:** 10.1002/alz.091465

**Published:** 2025-01-09

**Authors:** Samantha C. Burnham, Laura Harper, Tricia Locascio, Holly E McPherson, Michael Pontecorvo, Jian Wang

**Affiliations:** ^1^ Eli Lilly and Company, Indianapolis, IN USA; ^2^ Eli Lilly and Company, Indinapolis, IN USA

## Abstract

**Background:**

Presence of amyloid pathology is used to identify patients who would benefit from novel amyloid‐targeting therapies for Alzheimer’s disease (AD). Whilst PET tracers are considered the gold standard for establishing the presence of amyloid pathology, they are costly and not widely accessible. Blood‐based biomarkers, if interchangeable with PET, could help negate such an inequitable barrier to treatment. This study assesses the agreement and interchangeability between blood‐based biomarkers and amyloid‐PET.

**Method:**

BioHermes participants with MCI or AD‐dementia and available amyloid‐PET evaluations as well as blood‐based biomarker results for PrecivityAD (C2N) and/or P‐tau217 (research use MSD, Lilly) were included. Agreement (PPA, NPA, OPA, PPV, NPV) between amyloid‐PET and blood‐based biomarker stratification as well as the non‐inferiority (Figure 1) of blood‐based biomarkers to amyloid‐PET for patient identification were evaluated. All thresholds and methods for stratification were pre‐specified. Two‐threshold stratification for the blood‐based biomarkers were implemented.

**Result:**

537 participants with PrecivityAD and amyloid‐PET (mean‐age 73.2 years, 54.0% Female, 85.3% White, 47.3% amyloid‐PET positive) and 531 participants with P‐tau217 and amyloid‐PET (74.0 years, 53.9% Female, 85.3% White, 46.5% amyloid‐PET positive) were included. There was moderate to high agreement between the blood‐based biomarkers and amyloid‐PET visual read, Table 1. Higher agreement with amyloid‐PET visual read was achieved when participants between two‐thresholds were removed (and would require confirmatory CSF or PET evaluations). Generally, the P‐tau217 (MSD, Lilly) blood‐based biomarker demonstrated higher agreement with amyloid‐PET visual read in comparison to the Aβ blood‐based PrecivityAD test. Both blood‐based biomarkers, removing participants between the two‐thresholds, met criteria for non‐inferiority to amyloid‐PET clinical trial criteria for selecting patients with amyloid‐pathology, Table 2.

**Conclusion:**

A clinically available plasma test (PrecivityAD, C2N) as well as a research use P‐tau‐217 assay (MSD, Lilly) were both able to rule in patients that would also be selected by amyloid PET (PPV 86% and 88%, respectively). New plasma diagnostics that incorporate P‐tau217 or other more sensitive markers may be more accurate than Aβ blood‐based tests for identifying amyloid pathology. Results support the hypothesis that blood‐biomarkers may be interchangeable with amyloid‐PET criteria for selecting patients who are suitable for and would benefit from treatment with amyloid‐targeting therapies.